# Lithium carbonate in amyotrophic lateral sclerosis patients homozygous for the C-allele at SNP rs12608932 in *UNC13A*: protocol for a confirmatory, randomized, group-sequential, event-driven, double-blind, placebo-controlled trial

**DOI:** 10.1186/s13063-022-06906-5

**Published:** 2022-12-05

**Authors:** Sean W. Willemse, Kit C. B. Roes, Philip Van Damme, Orla Hardiman, Caroline Ingre, Monica Povedano, Naomi R. Wray, Marleen Gijzen, Mirjam S. de Pagter, Koen C. Demaegd, Annemarie F. C. Janse, Roel G. Vink, Boudewijn T. H. M. Sleutjes, Adriano Chiò, Philippe Corcia, Evy Reviers, Ammar Al-Chalabi, Matthew C. Kiernan, Leonard H. van den Berg, Michael A. van Es, Ruben P. A. van Eijk

**Affiliations:** 1 Department of Neurology, UMC Utrecht, Utrecht, The Netherlands; 2 Department of Health Evidence, Radboud UMC, Nijmegen, The Netherlands; 3 Department of Neurology, University Hospitals Leuven, Leuven, Belgium; 4 Department of Neurology, National Neuroscience Centre, Beaumont Hospital, Dublin, Ireland; 5 FutureNeuro SFI Research Centre, Royal College of Surgeons in Ireland, Dublin, Ireland; 6 Department of Clinical Neuroscience, Karolinska Institute, Stockholm, Sweden; 7 Functional Unit of Amyotrophic Lateral Sclerosis (UFELA), Service of Neurology, Bellvitge University Hospital, Hospitalet de Llobregat, Spain; 8 Institute for Molecular Bioscience, The University of Queensland, Brisbane, Australia; 9 Department of Genetics, UMC Utrecht, Utrecht, The Netherlands; 10 TRICALS, Utrecht, The Netherlands; 11 ‘Rita Levi Montalcini’ Department of Neuroscience, University of Turin, Turin, Italy; 12 Neurology, AOU Città della Salute e della Scienza Hospital of Turin, Turin, Italy; 13 Centre Constitutif SLA, CHRU de Tours - Fédération des centres SLA Tours-Limoges, LitORALS, Tours, France; 14 European Organization for Professionals and Patients with ALS (EUpALS) & ALS Liga Belgium, Leuven, Belgium; 15 Department of Basic and Clinical Neuroscience, King’s College London, Maurice Wohl Clinical Neuroscience Institute, London, UK; 16 Department of Neurology, King’s College Hospital, London, UK; 17 Department of Neurology, Royal Prince Alfred Hospital, Sydney, New South Wales Australia; 18 Brain and Mind Centre, University of Sydney, Sydney, New South Wales Australia; 19 Biostatistics and Research Support, Julius Centre for Health Sciences and Primary Care, Utrecht University, Utrecht, Netherlands

**Keywords:** Amyotrophic lateral sclerosis, Lithium carbonate, *UNC13A*, SNP rs12608932, Randomized controlled trial

## Abstract

**Background:**

Given the large genetic heterogeneity in amyotrophic lateral sclerosis (ALS), it seems likely that genetic subgroups may benefit differently from treatment. An exploratory meta-analysis identified that patients homozygous for the C-allele at SNP rs12608932, a single nucleotide polymorphism in the gene *UNC13A*, had a statistically significant survival benefit when treated with lithium carbonate. We aim to confirm the efficacy of lithium carbonate on the time to death or respiratory insufficiency in patients with ALS homozygous for the C-allele at SNP rs12608932 in *UNC13A*.

**Methods:**

A randomized, group-sequential, event-driven, double-blind, placebo-controlled trial will be conducted in 15 sites across Europe and Australia. Patients will be genotyped for *UNC13A*; those homozygous for the C-allele at SNP rs12608932 will be eligible. Patients must have a diagnosis of ALS according to the revised El Escorial criteria, and a TRICALS risk-profile score between −6.0 and −2.0. An expected number of 1200 patients will be screened in order to enroll a target sample size of 171 patients. Patients will be randomly allocated in a 2:1 ratio to lithium carbonate or matching placebo, and treated for a maximum duration of 24 months. The primary endpoint is the time to death or respiratory insufficiency, whichever occurs first. Key secondary endpoints include functional decline, respiratory function, quality of life, tolerability, and safety. An interim analysis for futility and efficacy will be conducted after the occurrence of 41 events.

**Discussion:**

Lithium carbonate has been proven to be safe and well-tolerated in patients with ALS. Given the favorable safety profile, the potential benefits are considered to outweigh the burden and risks associated with study participation. This study may provide conclusive evidence about the life-prolonging potential of lithium carbonate in a genetic ALS subgroup.

**Trial registration:**

EudraCT number 2020-000579-19. Registered on 29 March 2021.

**Supplementary Information:**

The online version contains supplementary material available at 10.1186/s13063-022-06906-5.

## Administrative information

Note: the numbers in curly brackets in this protocol refer to SPIRIT checklist item numbers. The order of the items has been modified to group similar items (see https://eur05.safelinks.protection.outlook.com/?url=http%3A%2F%2Fwww.equator-network.org%2Freporting-guidelines%2Fspirit-2013-statement-defining-standard-protocol-items-for-clinical-trials%2F&data=05%7C01%7CS.W.Willemse-9%40umcutrecht.nl%7Cedf62fc7c1cb4c2f974b08dabd62740f%7Cdcdf4a3dd0c04a6394cf781981249be5%7C0%7C0%7C638030527791755115%7CUnknown%7CTWFpbGZsb3d8eyJWIjoiMC4wLjAwMDAiLCJQIjoiV2luMzIiLCJBTiI6Ik1haWwiLCJXVCI6Mn0%3D%7C3000%7C%7C%7C&sdata=j1zicA4jDGupTz4CBG9vnzywd4M9RCj8%2F%2F4grQMPkc8%3D&reserved=0).

**Table:** 

Title {1}	Lithium carbonate in amyotrophic lateral sclerosis patients homozygous for the C-allele at SNP rs12608932 in *UNC13A*: protocol for a confirmatory, randomized, group-sequential, event-driven, double-blind, placebo-controlled trial
Trial registration {2a and 2b}.	EudraCT number 2020-000579-19. Registered on 29-03-2021.
Protocol version {3}	Version 1.5, 06-04-2022
Funding {4}	Funding was received from: MNDA, United Kingdom Fight MND, Australia FWO-Vlaanderen and ALS Liga, Belgium Stichting ALS Nederland, the Netherlands Ulla-Carin Lindquist Foundation, Sweden Thierry Latran Foundation, European Union Luzon Fundación, Spain My Name'5 Doddie Foundation, United Kingdom Alan Davidson Foundation, United Kingdom
Author details {5a}	Sean W. Willemse, Koen C Demaegd, Boudewijn THM Sleutjes, Leonard H. van den Berg, Michael A van Es, Ruben P.A. van Eijk, Department of Neurology, UMC Utrecht, The Netherlands Ruben P.A. van Eijk: Biostatistics and Research Support, Julius Centre for Health Sciences and Primary Care, Utrecht University, Utrecht, Netherlands Kit CB Roes: Department of Health Evidence, Radboud UMC, The Netherlands. Philip Van Damme: Department of Neurology, University Hospitals Leuven, Leuven, Belgium Orla Hardiman: Department of Neurology, National Neuroscience Centre, Beaumont Hospital, Dublin, Ireland, and FutureNeuro SFI Research Centre, Royal College of Surgeons in Ireland, Dublin, Ireland Caroline Ingre: Department of Clinical Neuroscience, Karolinska Institute, Stockholm, Sweden Monica Povedano: Functional Unit of Amyotrophic Lateral Sclerosis (UFELA), Service of Neurology, Bellvitge University Hospital, Hospitalet de Llobregat, Spain Naomi R Wray: Institute for Molecular Bioscience, The University of Queensland, Brisbane, Australia. Marleen Gijzen, Mirjam de Pagter, Department of Genetics, UMC Utrecht, The Netherlands. Annemarie FC Janse, Roel C Vink: Tricals, Utrecht, The Netherlands Adriano Chiò: ‘Rita Levi Montalcini’ Department of Neuroscience, University of Turin, Turin, Italy and Neurology, AOU Città della Salute e della Scienza Hospital of Turin, Turin, Italy Philippe Corcia: Centre Constitutif SLA, CHRU de Tours - Fédération des centres SLA Tours-Limoges, LitORALS, Tours, France Evy Reviers**:** European Organization for Professionals and Patients with ALS (EUpALS) & ALS Liga Belgium, Leuven, Belgium. Ammar Al-Chalabi: Department of Basic and Clinical Neuroscience, King's College London, Maurice Wohl Clinical Neuroscience Institute, London, UK and Department of Neurology, King's College Hospital, London, UK. Matthew C. Kiernan: Brain and Mind Centre, University of Sydney, Sydney, New South Wales, Australia and Department of Neurology, Royal Prince Alfred Hospital, Sydney, New South Wales, Australia.
Name and contact information for the trial sponsor {5b}	Stichting TRICALS Foundation Goeman Borgesiuslaan 77 3515 ET, Utrecht, The Netherlands Email: magnet@tricals.org
Role of sponsor {5c}	The sponsor is involved in collection, management, analysis, and interpretation of data; writing of the report; and the decision to submit the report for publication. Funders are not involved in the aforementioned activities.

## Introduction

### Background and rationale {6a}

Amyotrophic lateral sclerosis (ALS) is a rare, progressive neurodegenerative disorder leading to muscle weakness, behavioral symptoms, respiratory failure and, ultimately, death within on average three years after symptom onset [1, 2]. Patients with ALS are highly variable in their clinical presentation and progression rate, with survival times varying from a few months to over twenty years. The survival time after diagnosis is, on average, 17.7 months (95% CI 17.0–18.4) [3]. The lifetime risk of ALS is 1 in 300, with men being more susceptible than women, while prevalence remains limited with 3 to 5 cases per 100,000 people due to its poor prognosis [2, 4].

Riluzole, a glutamate release inhibitor, is currently the only licensed drug in Europe and extends the life expectancy by a few months [5]. Many different compounds have been investigated in order to expand the therapeutic options, but clinical trials have been mostly futile due to a variety of reasons [6, 7]. Over the last decade, it has become increasingly evident that ALS may not be a single disease, but could be considered a collection of disorders with clinically similar phenotypes yet variable underlying pathophysiological pathways and genetic mutations [1].

It seems unlikely, therefore, that we will be able to treat all forms of ALS with the same single drug. The heterogeneity in treatment response was confirmed in an exploratory meta-analysis of 606 patients treated with lithium carbonate [8]. Overall, no benefit to survival was observed in the general ALS population, but a subgroup of patients homozygous for the C-allele at SNP rs12608932 in *UNC13A* seemed to benefit of lithium carbonate. Lithium carbonate is an approved drug which is used extensively in the treatment of psychiatric illnesses, such as bipolar disorder, depression and psychosis [9–11]. It has been shown to influence many pathways, including the induction of sprouting of pyramidal neurons in the corticospinal tract and the promotion of synaptogenesis, and plays a role in autophagy [12]. All these mechanisms are potentially relevant to ALS, whereas the *UNC13A* protein is involved in synaptic vesicle maturation and neuronal outgrowth [13].

In this study, we aim to validate the exploratory findings of the meta-analysis and confirm the potential of lithium carbonate to prolong the time to death or respiratory insufficiency in patients with ALS homozygous for the C-allele at SNP rs12608932 in *UNC13A.* We will initiate a group-sequential, placebo-controlled, event-driven design to generate confirmatory evidence and minimize the exposure of patients to potentially ineffective treatment arms. In previous controlled clinical trials, lithium carbonate was relatively well tolerated with a mild safety profile [14, 15].

### Objectives {7}

The primary objective of this study is to assess the efficacy of lithium carbonate versus placebo on the composite endpoint of respiratory support free survival, defined as death from any cause or respiratory insufficiency in patients with ALS.

Secondary objectives of this study include the effect of lithium carbonate versus placebo on a combined assessment of mortality and measures of daily functioning, the revised Amyotrophic Lateral Sclerosis Functional Rating Scale (ALSFRS-R), respiratory function, plasma creatinine, and the time to reach advanced disease stages. Further secondary objectives are to evaluate the safety and tolerability of lithium administered orally to patients with ALS, and to assess the effect of lithium carbonate versus placebo on change in urinary P75^ECD^, in plasma neurofilament light and heavy chain, on cognitive functioning and on quality of life.

### Trial design {8}

This is a phase III, event-driven, double-blind, randomized, placebo-controlled, group sequential study to evaluate the efficacy of lithium carbonate in patients with ALS homozygous for the C-allele at SNP rs12608932. Assuming a prevalence of 14.21%, approximately 1200 patients will be genotyped in order to enroll 171 patients. Patients will be randomized to receive either lithium carbonate or placebo in a ratio of 2:1, resulting in 114 patients on lithium carbonate and 57 on placebo. To minimize the burden for patients on placebo, we defined an early stopping rule for both futility and superiority.

## Methods: participants, interventions, and outcomes

### Study setting {9}

This study will be conducted in 14 University Medical Centers in Australia, Spain, the UK, Belgium, Sweden, and The Netherlands. A list of participating centers can be found in the Supplementary material.

### Eligibility criteria {10}

A patient must meet all of the following criteria to be eligible to participate in this study:

1. Age ≥ 18 years at the time of screening.
2. Diagnosis of ALS according to the revised El Escorial criteria (possible, probable- laboratory supported, probable or definite) [16].
3. Capable of providing informed consent and complying with trial procedures, including randomization to sub-studies.
4. TRICALS risk profile > -6.0 and < −2.0**.
5. The use of riluzole will be permitted during the study. Patients taking riluzole must be on a stable dose for at least 30 days prior to the baseline visit or stopped taking riluzole at least 30 days prior to the baseline visit.
6. Women of childbearing potential, i.e., following menarche and until becoming post-menopausal unless permanently sterile, must have a negative pregnancy test at baseline and be non-lactating.
7. Men must agree to practice contraception for the duration of the trial and for at least 3 months after last dose of study drug.
8. Men must not plan to father a child or to provide sperm for donation for the duration of the trial and 3 months after the last dose of study drug.
9. Women must not be able to become pregnant (e.g., post-menopausal (i.e., no menses for 12 months without an alternative medical cause), surgically sterile or using effective birth control methods) for the duration of the study. Effective contraceptives are defined as having a failure rate of less than 1% per year when used consistently and correctly and, when applicable, in accordance with the product label, including: abstinence, hormonal contraception, intrauterine device in place for ≥ 3 months.

** The TRICALS risk profile is estimated using an open-access calculator, and is based on seven clinical disease characteristics [17]. The TRICALS risk profile can be conceptualized as a relative summary of prognostic information and is a predictor of the disease progression rate during follow-up [18]. The risk profile indicates how patients compare to each other (i.e., who is faster or slower progressing than average) without estimating the absolute survival time or probability. It is based on the ENCALS survival model, a cross-validated model to predict the composite endpoint death or respiratory insufficiency based on data of 11,475 patients with ALS [19]. The eligibility window was determined using clinical trial data from six independent trial cohorts comprising 1028 patients with ALS [18]. We searched for the optimal eligibility window that resulted in the most homogenous trial population in terms of ALSFRS-R progression without exceeding an exclusion rate of 25.0%.

A potential patient who meets any of the following criteria will be excluded from participation in this study:

1. Patients heterozygous or homozygous for the A-allele at rs12608932 (*UNC13A*).
2. Safety Laboratory Criteria at baseline:
  ALT ≥ 5 times upper limit of normal (ULN)
  AST ≥ 3 times ULN
  Bilirubin ≥ 1.5 times ULN
  Plasma sodium <120 mmol/L
  Creatinine clearance < 50 mL / min (Cockcroft-Gault) based on Cystatin C; if not available, eGFR can also be calculated based on creatinine clearance.
  Platelet concentration of < 100 × 109 per L
  Absolute neutrophil count of < 1 × 109 per L
  Hemoglobin < 100 g/L (<6.2 mmol/L)
  Amylase and lipase ≥ 2 times ULN (suspected pancreatitis)
  Lactate ≥ 2 times ULN (suspected lactate acidosis)
3. Moderate to severe hepatic impairment according to Child-Pugh classification (Class B or higher; score ≥ 7). Child-Pugh classification is based on bilirubin, albumin, International Normalized Ratio (INR) and presence of encephalopathy or ascites.
4. Participation in any other investigational drug trial or using investigational drug (within 30 days prior to screening).
5. Hypothyroidism unresponsive to thyroid hormone supplementation.
6. Patients using non-invasive ventilation (NIV, ≥22 h per day) or having a tracheostomy.
7. Patients taking edaravone within 30 days prior to screening. Edaravone is approved by the FDA, but remains an investigational product in Europe and Australia.
8. Clinically significant history of unstable or severe cardiac (e.g., congestive heart failure, coronary insufficiency and arrhythmias), oncological, hepatic or renal disease, neuromuscular diseases, significant pulmonary disorder or other medically significant illness.
9. Drug or alcohol abuse.
10. Bipolar disorder, unstable psychiatric illness defined as psychosis or untreated major depression within 90 days of the screening visit. This exclusion criterion is based on a prior psychiatric diagnosis that is unstable as determined by the patient’s treating psychiatrist.
11. Presence of frontotemporal dementia which prevents informed consent.
12. Known allergy or hypersensitivity to lithium, or its excipients, or to the components of the placebo.
13. Brain injury with posttraumatic epilepsy or neurologic deficit, excluding a concussion in the medical history. Brain infarction is an exclusion criterion, a transient ischemic attack is not.
14. Addison disease.
15. Patients with the following co-medication: antipsychotics, digoxin and calcium antagonists, carbamazepine, methyldopa, verapamil and diltiazem.
16. Brugada syndrome or family history of Brugada syndrome.

### Who will take informed consent? {26a}

Prior to performing any study-related activities under this protocol, including screening tests and assessments, written informed consent (IC) will be obtained from the patient. The patient information will be provided prior to the screening visit. A trial physician will conduct the IC process and inform patients on the details of the study. The trial physician will sign the informed consent after the patient indicates that he or she has been given enough time to consider participation, and when all questions are adequately answered. A copy of the IC, signed and dated by the patient and trial physician, is given to the patient. Confirmation of a patient’s IC will be documented in the patient’s medical record.

### Additional consent provisions for collection and use of participant data and biological specimens {26b}

An option for permission to reuse clinical data and any blood and urine not fully used in the current study is included in the IC form. If patients have given consent to perform a lumbar puncture, the same applies to any remaining cerebrospinal fluid.

## Interventions

### Explanation for the choice of comparators {6b}

A matched placebo will be used. The use of a placebo group provides us the ability to distinguish between effects directly caused by the active drug and those effects resulting from variation in disease progression. Optimal standard of care will be offered to all patients in addition to study medication. Changes in care after randomization (e.g., stop of Riluzole, gastrostomy placement, or use of non-invasive ventilation) are permissible and will be registered as potential intercurrent events.

### Intervention description {11a}

Lithium salts such as lithium carbonate or citrate have been used for the treatment of bipolar disorder for decades for its mood stabilizing properties [9–11]. Within this population, lithium carbonate has been used effectively and safely with adequate monitoring. As such, its side-effects, both short- and long term, are well known. Given the fact that multiple trials with lithium carbonate have been performed in ALS, the side-effects of lithium carbonate in the target population of this study are also well known [14, 15, 20].

The study drug will be produced by TioFarma BV and is available as 400 mg capsules of lithium carbonate or matched placebo. Both the study drug and placebo are matched in appearance and packaging, and taken as capsules. Capsules will be taken once daily, starting with one capsule (400 mg daily) initially titrated up to two or three capsules daily over the first four weeks of treatment, depending on blood lithium levels. Blood levels will be measured at baseline, 7 days (12 h +/− 30 min after previous evening dose), and at 14 days (12 h +/−30 min after previous evening dose). The target range for the lithium plasma level will be between ≥0.4 mmol/l and ≤0.8 mmol/l. Further plasma level measurements will be taken at 21 and 28 days to confirm the target range has been achieved. Ongoing lithium level monitoring will be scheduled for week 8 and week 12, and quarterly thereafter for the duration of the study. Dose adjustments can be made by reducing back to 2 capsules daily or to 1 capsule daily as required. It is anticipated that most patients will remain on 2 capsules throughout the duration of the trial. Sham dose adjustments will also be made to patients on placebo to maintain blinding. Unused drug tablets and packaging will be returned during each visit.

Lithium plasma levels will be stored in the centralized online system (Research Online and Castor). Lithium plasma levels are sent to a dedicated mailbox for review by unblinded physicians. They will enter the new dose in the eCRF; subsequently the patient and the site staff will be notified. Study personnel should verify with the patient (by email or phone) that the dose adjustments resulting from the day 7 and day 28 lithium blood draw have been received and understood. On average the lithium levels will be stable after approximately three to four weeks (or 3–4 blood samples).

The Lithium Side Effects Rating Scale (LiSERS) will be used to monitor lithium use and flag potential lithium intoxications [21]. The LiSERS questionnaire may be completed by either the patient via email, or by study personnel via phone at days 7, 14, 21, 28, and 56 and after 3 months (within a 5-day window) since randomization. If lithium intoxication is suspected, the treating physician and/or unblinded physician are contacted to take appropriate action; dose-adjustments may be necessary.

### Criteria for discontinuing or modifying allocated interventions {11b}

Patients can withdraw from the trial at any time. Dose changes are made according to the plasma lithium levels which are determined regularly at the start of the trial. Furthermore, the LiSERS questionnaire will be used to monitor side effects and possible lithium intoxications which would warrant changes in dosage. In the exceptional case when plasma lithium levels exceed 0.8 mmol/l on two consecutive occasions while on the minimum dose of 1 capsule per day (400mg per day), this will be accepted if concentrations remain ≤1.0 mmol/l (for patients 65 years or older) or ≤1.5 mmol/l (for patients ≥18–<65 years). If values exceed these limits the investigational product must be permanently discontinued. Additionally, when patients display symptoms of a lithium intoxication (as evaluated by the unblinded physician and based on changes in LiSERS questionnaire outcome), the investigational product will also be permanently discontinued.

Interventions will not be withdrawn in case of worsening clinical condition due to ALS.

Patients will be withdrawn from the study by the Investigator in consultation with the coordinating Investigator and the Sponsor in case of:

1. Liver toxicity where one or more of the following criteria (in alignment with the FDA premarketing clinical liver safety guidance) are met and no compelling alternate cause is):
  1. ALT ≥ 3×ULN and bilirubin ≥2×ULN (>35% direct bilirubin)
  2. ALT ≥ 5×ULN
  3. ALT ≥ 3×ULN and with symptoms believed to be related to liver injury or hypersensitivity.
  4. ALT ≥ 3× baseline ALT and with symptoms believed to be related to liver injury or hypersensitivity.
2. Allergic reaction criteria are met and no compelling alternate cause is identified:
  ° CTCAE Grade III/IV: severe skin reaction (Steven-Johnson syndrome, Toxic Epidermal Necrolysis or Erythema Multiforme). Patients with Grade ≥3 allergic reactions that are considered to be possibly or probably related to the investigational product should permanently discontinue the investigational product regimen and the patient should be withdrawn from the study. Patients should be treated as clinically appropriate and followed until resolution of the adverse event.
  ° Patients may continue investigational product for CTCAE Grade I or II allergic reactions at the discretion of the Investigator. The patient should be advised to contact the Investigator immediately if there is any worsening of symptoms or if further systemic signs or symptoms develop. Antihistamines, topical corticosteroids, or antipruritic agents may be prescribed.
  ° If a profuse, purpuric and coalescing leukocytoclastic vasculitis occurs, the patient should be withdrawn from the study.
3. Development of moderate to severe renal impairment as measured by Cystatin C (< 50 mL/min).
4. Note: In case of renal impairment, medication may be stopped for 2 weeks, after which the patient is retested. If the renal impairments persist, patients are withdrawn from the study.
5. The patient becomes pregnant.
6. Noncompliance with the protocol as judged by the investigator.

### Strategies to improve adherence to interventions {11c}

Lithium blood plasma levels will be checked during each visit to ascertain therapy adherence. Patients will be phoned after they receive a dose change to improve adherence. To minimize the burden for patients, Blood samples are analyzed at the local lab and results are sent to the unblinded physicians for dose adjustment review. If a local option is not feasible, patients are requested to visit their study site.

### Relevant concomitant care permitted or prohibited during the trial {11d}

Patients taking concomitant riluzole at study entry must be on a stable dose for at least 30 days prior to the baseline visit and must continue taking the same dosage throughout the study, unless the Investigator determines that riluzole should be discontinued for medical reasons, in which case it may not be restarted. Concomitant medication for symptom management (i.e., spasms or saliva management) during the study is at the discretion of the Principal investigator. Daily intake of all concomitant vitamins and supplements should, preferably, be stabilized at least 30 days prior to the baseline visit.

If a study patient initiates Edaravone treatment during the study, the patient will be discontinued from the study treatment. Additionally, any investigational therapy being evaluated for efficacy in ALS or other diseases is prohibited beginning 30 days prior to the baseline visit and throughout the study. Use of antipsychotics (e.g., haloperidol, thioridazine, fluphenazine, chlorpromazine, olanzapine and clozapine), digoxin, calcium antagonists, carbamazepine, methyldopa, verapamil and diltiazem are prohibited during the study due to their interactions with lithium carbonate.

### Provisions for ancillary and post-trial care {30}

After completion of the 24-month follow-up period, patients will be offered to enroll in an open-label extension phase. Possible damages caused by participation in the current trial are covered by an insurance taken out by the Sponsor in accordance with legal requirements. The insurance applies to the damage that becomes apparent during the study or within 4 years after the end of the study.

### Outcomes {12}

#### Primary outcomes

The primary endpoint of this study is a composite endpoint, defined as time to death from any cause or respiratory insufficiency (tracheostomy or the use of non-invasive ventilation for ≥22 h per day for ≥10 consecutive days).

#### Secondary outcomes

1. Combined effect on daily functioning and overall survival based on a joint model of survival and ALSFRS-R total scores.
2. Daily functioning, defined as mean change from baseline in ALSFRS-R total score. Disease progression will be monitored using the ALSFRS-R, which is a validated 12-item questionnaire designed to evaluate the functioning of respiratory, bulbar, fine and gross motor function in ALS patients [22]. The total score ranges from 48 (no limitation in daily activities) to 0 (total inability). The ALSFRS-R slope, the difference in scores standardized for time, is often used as measure of disease progression in clinical trials and is significantly associated with survival [23].
3. Respiratory function, defined as mean change from baseline in Slow Vital Capacity (SVC). The SVC is associated with survival in ALS patients [24]. The obtained estimates of the vital capacity will be recorded in liters and standardized using the GLI-2012 reference standard [25].
4. Change in outcome of Plasma creatinine. Plasma creatinine is a part of the safety tests and will be used as one of the secondary endpoints to monitor disease progression [26]. Plasma creatinine is well correlated with functional decline, muscle strength and is highly predictive for overall survival. Due to the effect of disease progression on plasma creatinine, Cystatin C will be used for monitoring kidney function as safety parameter [27].
5. Clinical disease stage, defined as mean time spent in each stage of the King’s and ALS Milano-Torino staging systems. The King’s staging system is a simple clinical staging system, which defines four stages of ALS. The first three stages are defined by functional involvement of a region: bulbar, upper limbs and lower limbs. The number of regions involved gives the stage. Stage 4 is reached if swallowing (4A) or respiratory (4B) difficulty is severe enough to require intervention. This procedure takes the form of a semi-structured interview. MITOS is a similar clinical staging system, in which the stages are defined as loss of independence on the four domains of the ALSFRS-R: swallowing, walking/self-care, communicating and breathing [28]. Studies have shown that both King’s staging and the ALS-MITOS correlate with survival [29, 30].
6. Safety based on the safety assessments including neurological examinations, clinical laboratory evaluations, vital signs and frequency of adverse events (AEs) or serious adverse events (SAEs). (S)AEs will be categorized according to the Common Terminology Criteria for Adverse Events and will be rated for severity and association with study drug.
7. Tolerability defined as time-to-discontinuation of assigned treatment since randomization.
8. Change from baseline in urinary P75^ECD^. P75 is a neutrotrophin receptor that is expressed after motor neural damage and excreted through the urine [31]. Hence, presence of P75^ECD^ in the urine is indicative for the degeneration of motor neurons. P75^ECD^ has been shown to increase over time in patients with ALS and correlates well with measures of functional decline. Moreover, the marker is associated as independent predictor of survival. Therefore, P75^ECD^ may have the utility as a potential pharmacodynamic biomarker and slowing of a rising urine level may be indicative of a biological effect.
9. Change from baseline in plasma Neurofilament light and heavy chain. Neurofilament light and heavy chains in ALS are well known to be correlated with prognosis and remain relatively stable over time [32]. A change in the expression of neurofilament light and/or heavy chain may, therefore, be considered as supporting evidence of disease amelioration in ALS.
10. Neuropsychological status, defined as change from baseline on the ECAS and ALS-FTD-Q. Approximately 35% of the patients, with ALS, experience cognitive or behavioral impairment. The cognitive and behavioral impairment may increase as disease progresses [33]. The neuropsychological status of patients will be evaluated using the Edinburgh Cognitive and Behavioral ALS Screen (ECAS) [34]. The ECAS is independent of motor disability and evaluates five cognitive domains: language functions, executive functions, and letter fluency, memory and visuospatial functioning. If available, ECAS version A and B will be alternated to prevent a training effect in patients. The questionnaire has a behavior screen with a carer interview, this part will not be performed in this study. In addition, we will evaluate behavioral disturbances using the ALS frontotemporal dementia questionnaire (ALS-FTD-Q) [35]. The ALS-FTD-Q is a validated instrument for the screening of behavioral disturbances in ALS. The ALS-FTD-Q has 25 items (including three cognitive items: memory, concentration, and orientation in time), with a 4-point rating scale; the maximum score is 100. A higher score indicates more behavioral disturbances.
  Global quality of life (QoL), defined as change from baseline on the visual analog scale (single-item scale) and EQ-5D. QoL will be measured using a visual analog scale (single-item scale): a horizontal line of 100 mm ranging from 0 (worst imaginable QoL) to 100 (perfect QoL) [36]. Generic QoL will be measured using the EQ-5D. The EQ-5D measures the health status in terms of five dimensions: mobility, self-care, usual activities, pain and depression. From the EQ-5D a quality-adjust life year (QALY) can be calculated, which is required for cost-utility analysis if a drug is beneficial for patients with ALS [37]. If a patient deceases during the follow-up period, the date will be recorded. If a patient reaches the criteria for respiratory insufficiency (tracheostomy or the use of non-invasive ventilation for ≥22 h per day for ≥10 consecutive days), the date this criterion was met will be recorded and the patient will no longer be part of the study. The time point for all secondary outcomes is month 24.

#### Exploratory outcomes

The effect of lithium carbonate on motor nerve function will be assessed as an exploratory objective. This procedure will only be performed at the UMC Utrecht (the Netherlands). A compound muscle action potential (CMAP) scan or electrophysiological muscle scan (MScan) is a non-invasive, well-tolerated electrophysiological method that provides information on the number of motor units, their size and axonal excitability [38–43]. The CMAP scan records the electrical muscle activity (CMAP) using surface electrodes. Approximately 500 transcutaneous nerve stimuli are delivered with decreasing stimulus currents ranging from supramaximal to subthreshold levels. The CMAP scan quantifies progression in muscles affected by motor neuron disease, and is related to functional decline and survival [38, 44–46] We will apply the CMAP scan in the lower arm (abductor pollicis brevis and abductor digiti minimi) and lower leg (extensor digitorum brevis and abductor hallucis, if the extensor digitorum brevis is paralytic, the tibialis anterior will be used), both left- and right-sided, at quarterly intervals during the first 12 months of follow-up.

### Participant timeline {13}

The participant timeline can be seen in (Table 1). Study medication administration and concomitant medication or medical procedures are continuously recorded. Adverse events are continuously monitored and documented. Serious adverse events are continuously monitored and reported.

**Table 1:** Participant timeline

	Screening (−45 days)	Baseline	Days 7, 14, 21, 28, and 56	Month 3 (±7 days)	Month 6 (±7 days)	Month 9 (±7 days)	Month 12 (±7 days)	Month 15 (±7 days)	Month 18 (±7 days)	Month 21 (±7 days)	Month 24 (±7 days)
Informed consent	X										
Eligibility criteria review	X	X									
DNA sample (10 mL)^a^	X										
Randomization		X									
Demographic data and medical history	X	X									
Neurological examination		X					X				X
12-lead ECG		X		X			X				X
Dispense study medication		X		X	X	X	X	X	X	X	
Drug accountability and compliance				X	X	X	X	X	X	X	X
Vital signs and body weight		X		X	X	X	X	X	X	X	X
Blood sample (30 ml)		X		X	X	X	X	X	X	X	X
Blood sample (lithium level)			X	X	X	X	X	X	X	X	X
Urine sample (20 ml)		X		X	X	X	X	X	X	X	X
Pregnancy test^b^	*For WOCBP patients: at a monthly interval*										
Spirometry (SVC)	X	X		X	X	X	X	X	X	X	X
ALSFRS-R	X	X		X	X	X	X	X	X	X	X
Quality of life (VAS and EQ-5D)		X		X	X	X	X	X	X	X	X
LiSERS questionnaire			X	X							
King’s staging system		X		X	X	X	X	X	X	X	X
ECAS		X					X				X
ALS-FTD-Q		X					X				X
Optional: liquor biomarker sample		X			X		X				
Optional: CMAP scan^c^		X		X	X	X	X				
Survival data and ALS interventions^d^	X	X		X	X	X	X	X	X	X	X

^a^End-of-Study or early-termination visit

^b^Pregnancy tests can be performed by the patient themselves at home

^c^CMAP scan is only performed in the UMC Utrecht

^d^ALS interventions are gastrostomy, non-invasive ventilation, wheelchair use and tracheostomy

#### Sample size {14}

The sample size calculation for this event-driven trial is based on the primary efficacy end point death of any cause or respiratory insufficiency. The trial continues until the required number of events is reached or until 24 months after the last enrolled patient, whichever comes first. In the meta-analysis of lithium carbonate among patients homozygous for the C-allele at SNP rs12608932 in *UNC13A*, we found a crude hazard ratio (HR) of 0.43 and an adjusted HR of 0.30 (active vs. placebo) [8]. Due to the relatively small sample size of the meta-analysis (*n* = 46), we assumed a conservative hazard ratio of 0.50. With the use of a one-sided test at the 0.0250 significance level, the trial would have 80% power if it continued until 72 events occurred in the combined trial groups. We specified one pre-specified stopping rule for efficacy based on the Lan- DeMets procedure with O’Brien-Fleming alpha-spending function when 41 events occur (non-binding).

We used a simulation-based approach to estimate the event probability during follow-up and the required sample size. We assumed that 14.21% of the patients will be homozygous for the C-allele at SNP rs12608932 in *UNC13A*. With a planned maximum enrolment rate of 5–6 patients per month, enrolment of 171 patients (114 on active treatment and 57 on placebo) resulted in 72 events within 48 months since first enrolled patient for 99.5% of our simulated trials.

#### Recruitment {15}

Patients fulfilling the inclusion criteria, apart from homozygosity for the C-allele at SNP rs12608932 in *UNC13A* will be identified by their treating physicians and given the patient information verbally and handed the written information for review. Alternatively, patients can register themselves via the TRICALS website, after which patients will be approached with information regarding the trial [47]. Afterwards, a screening visit will be planned where informed consent will be taken and a blood sample for rs12608932 genotyping, or consent to extract the rs12608932 genotype if already available in other sources.

## Assignment of interventions: allocation

### Sequence generation {16a}

Randomization will be done using a centralized online response system (Research Online, Utrecht, the Netherlands, and Castor EDC, Amsterdam, The Netherlands), which assigns each randomized participant a unique identification number which will be used throughout the study. Randomization sequences in the study will be in random block sizes of 3–9, and stratified for trial site and risk category (high vs. low risk; low risk defined as a TRICALS risk profile of ≤ −4.5).

### Concealment mechanism {16b}

The allocation sequence is concealed by the centralized online response system.

### Implementation {16c}

Implementation is performed by research nurses and trial physicians.

## Assignment of interventions: blinding

### Who will be blinded {17a}

All staff, patients and personnel involved with the study will be masked to treatment, except site pharmacy personnel and the unblinded physicians. Furthermore, at least one sponsor safety employee will be unblinded in the case of serious safety events requiring unmasking. Information on lithium plasma levels is only accessible by the unblinded physicians. Lithium plasma levels will not be reported in (electronic) patient records.

### Procedure for unblinding if needed {17b}

In a medical emergency when identifying the patient’s treatment assignment may influence the patient’s clinical care, the Investigator may request the patient’s treatment assignment with the unblinded physician; 24-h emergency unblinding is available through the local pharmacy.

## Data collection and management

### Plans for assessment and collection of outcomes {18a}

During each clinical assessment patients will be asked about their current medication use and potential (serious) adverse events. All prescription drugs, over-the-counter medications, nutraceuticals, and herbal remedies will be recorded in the electronic case report form (eCRF). Additionally, ALS specific interventions such as gastrostomy placement, recommendation for wheelchair- and non-invasive ventilation (NIV) use will be recorded. Basic vital signs measurements (systolic and diastolic blood pressure, respiratory rate, heart rate and temperature) and body weight will be obtained. Details of all pregnancies in female patients and, female partners of male patients will be collected after the start of study drug and until conclusion of the pregnancy.

The ALSFRS-R questionnaire, the King’s- and ALS-MITOS staging will be filled out. Lung function is evaluated by using a non-invasive spirometer of the brand EasyOne to determine the Slow Vital Capacity (SVC), of which the highest of three attempts will be registered. During each follow-up period a venipuncture will be performed; approximately 20 ml will be obtained (4 tubes). All safety assessment will be evaluated locally. At baseline, Beta – HCG will be determined for female study patients. Additionally, patients will be asked to provide a urine sample. The urine sample (20 ml) will be used to screen for safety (dipstick or other method) and pregnancy.

Patients can optionally partake in lumbar punctures and CMAP scans. During the lumbar puncture, one vial (approximately 5 mL) of cerebrospinal fluid will be obtained at baseline, month 6 and month 12. Blood (serum and plasma), urine and CSF collected for biomarkers will be stored locally at −80 °C for at least the duration of the trial. CMAP scans will be made at baseline and months 3, 6, 9, and 12.

### Plans to promote participant retention and complete follow-up {18b}

As ALS progresses, patients may experience increasing difficulty to adhere to the nominal visiting schedule. To reduce discontinuations due to non-compliance, we provide an alternative monitoring option for debilitated patients, if allowed according to local laws and regulations. If, in the judgment of the principal investigator, the patient cannot reasonably be expected to travel to the clinic, the minimum data required for continued participation in the study (i.e., remain on the study medication) are laboratory tests, adverse event reporting and ALSFRS-R, all of which can be obtained remotely by a trial nurse, either during a home visit or by phone. Blood samples for safety assessments may be obtained by the patient’s general practitioner or any other local blood collection option that has been approved by the Sponsor. These data are to be obtained according to the nominal visit schedule until the patients withdraws, reaches the endpoint or the study completes, whichever comes first. If the patient becomes unfit for travel, this information will be documented in the source data. If no accommodations can be made for adequate safety monitoring, patients are withdrawn from study medication and the minimum requirement for follow-up will be assessment of survival data (i.e., living status or date of death).

### Data management {19}

A data management plan is available for this study. In brief, at screening the centralized online system will assign a unique patient identification number to each participant (Research Online, Utrecht, the Netherlands and Castor EDC, Amsterdam, The Netherlands). This identification number contains three letters to identify the site and three numbers to identify the patient (e.g., for the first patient in the UMC Utrecht; UMC-001). The identification number will be used for all eCRFs, laboratory tests and other administrative documents. Any patient identification numbers that are assigned will not be reused, even if the patient does not receive treatment (e.g., screening failures). All study data will be collected in pseudonymized form and stored in the online database of Research Online, located at the server of the UMC Utrecht, and Castor ED, located in the Netherlands. Appropriate technical and organizational measures will be implemented in order to ensure compliance with the GDPR. The trial nurse(s), research physician(s) and study monitors have access to this database. The data will be kept for 25 years.

### Confidentiality {27}

All data sheets are secured with a password and therefore only accessible for authorized individuals. The data will be saved on the server of the UMC Utrecht or the server of Castor. In case of inspection by health authorities (i.e., high rates of SUSARs) the study staff is obligated to provide the individual data. Patients are informed about this possibility by the patient information letter.

### Plans for collection, laboratory evaluation and storage of biological specimens for genetic or molecular analysis in this trial/future use {33}

Genetic testing will be performed for one genetic variant; the C-allele at SNP rs12608932. A blood sample of 10mL will be collected. DNA samples will be shipped to UMC Utrecht, Utrecht, the Netherlands (European samples) or Queensland Brain Institute, Brisbane, Australia (Australian samples)*.*

## Statistical methods

### Statistical methods for primary and secondary outcomes {20a}

The number and percentage of patients screened, randomized, receiving any study medication, completing the study, prematurely discontinuing treatment, and prematurely discontinuing from the study will be summarized by treatment arm and overall participation. Reason for premature discontinuation will be summarized.

#### Demography and baseline characteristics

Continuous variables are expressed as mean and standard deviation for normally distributed variables and median and range for non-normally distributed variables. Categorical variables will be presented as number of cases and percentage. Baseline characteristics will be compared between the placebo and intervention arm without significance testing. Demography will be summarized by allocation arm (active *vs.* placebo) and overall. Missing data will not be imputed unless otherwise specified. The following baseline variables will be calculated:

- Body mass index = weight (kg)/height^2^ (m)
- SVC _Predicted_ (L) = Predicted using GLI equation (Age, height, ethnicity, sex)
- Predicted %SVC = [SVC _Observed_ (L)/SVC _Predicted GLI2012_ (L)]*100% [48]
- Diagnostic delay (months) = [date diagnosis – date symptom onset]/365.25
- Symptom duration (months) = [date randomization – date symptom onset]/365.25
- ΔFRS = [ALSFRS-R _Baseline_ – 48]/symptom duration [49]
- TRICALS risk category: quintiles of the linear predictor based on the model described in Westeneng et al. [19]. Linear predictor is based on age, diagnostic delay, %SVC _Baseline_, ΔFRS, FTD, site of symptom onset, and El Escorial classification (see also {10} Eligibility criteria).
- Utrecht stage: Based on the ENCALS model, the median survival time from onset is predicted per patient (in months). Utrecht Stage is defined as symptom duration/median predicted survival time. Staging categories are I = 0–0.25; II = 0.25–0.5; III = 0.5–0.75; and IV = >0.75.

#### Analysis populations

- *The efficacy population* will be used for the primary efficacy analysis. The efficacy population includes all randomized patients irrespective of follow-up or receiving the actual study medication (intention-to-treat). Patients will be analyzed according to their randomization assignment.
- *The safety population* will consist of all randomized patients who received at least one dose or portion of a dose of study medication. Patients will be analyzed according to the treatment they received.

#### General methods

The primary endpoint hypothesis tests will be 2-sided with a significance level of *α* = 0.05. Secondary endpoints will be considered exploratory, presented with 95% confidence intervals and without inferential statements. For each analysis, all available data (from first date of randomization to last follow-up) will be used.

#### Statistical methods for primary outcome

The primary endpoint of this study is a composite of time to death from any cause or respiratory insufficiency (DRI; defined as tracheostomy or the use of non-invasive ventilation for ≥22 h per day for ≥10 consecutive days). For each individual we will calculate the primary endpoint as [date of event/censor – date of randomization]/365.25. An indicator variable will be defined specifying whether the patient was alive or had an event at the end of the study. To test the difference between treatment arms, we will use a likelihood ratio test stratified for the randomization factor “*trial site*” and adjusted for “*TRICALS Risk Profile*” as continuous variable. The TRICALS Risk Profile will be fitted as linear effect. This likelihood ratio test will also be used for sequential monitoring of efficacy during one pre-specified interim analysis. The Kaplan-Meier plots estimating the cumulative event rate will be provided for each treatment group. In case of non-proportional hazards, we will estimate the mean difference in Restricted Mean Survival Time (RMST).

#### Statistical methods for secondary outcomes

I Combined assessment of daily functioning and survival, defined as death from any cause [50, 51]. A joint model will be constructed between a survival model and a longitudinal model for ALSFRS-R data to evaluate the combined effect on both endpoints simultaneously; the full testing procedure is described elsewhere [52]. In short, the survival sub-model will contain treatment allocation as covariate and will be stratified for randomization factors “trial site and adjusted for “TRICALS Risk Profile” as a continuous variable, where the baseline hazard function follows a Weibull distribution [53]. A mixed effects sub-model will be fitted for the ALSFRS-R data with a random intercept and random slope for time per individual. The fixed effects part contains: baseline ALSFRS-R, treatment, time, baseline ALSFRS-R*time and treatment*time. Time will be fitted both linearly as well as non-linearly using restricted cubic splines. The non-linear effect of time will be kept in the model only if significant. If not, fixed non-linear time will be deleted first, followed by random non-linear time. An unstructured covariance matrix will be used to model the correlations among repeated measures. A likelihood ratio test will be used to compare a joint model with the treatment terms vs. a joint model without the treatment terms, thereby estimating the combined effect on both the ALSFRS-R and mortality, irrespective of their association.
II ALSFRS-R scores will be analyzed using the same joint model framework outlined above to account for the informative censoring due to patient death (i.e., mortality-adjusted progression). Here, we will use a likelihood ratio test to evaluate the time*treatment interaction from the ALSFRS-R sub-model. A similar strategy will be applied for change from baseline in Predicted %SVC, plasma creatinine, and QoL.

#### Efficacy: other study parameters

III Time-to-recommended wheelchair utilization. For each individual, we will define an indicator variable whether the patient uses (or has a recommendation for) a wheelchair and calculate the time from randomization to wheelchair use or censoring. The endpoint will be analyzed as described for the primary analysis.
IV Time spent in disease stage. Based on of the King’s, ALS Milano-Torino and Utrecht staging systems we will calculate the mean time spend in each stage during follow-up. The mean time will be compared between treatment arms.
V Change from baseline in Urinary P75^EDC^. The change from baseline in Urinary P75^EDC^ will be analyzed using a mixed effects model as described under II.
VI Change from baseline in Neurofilament light and heavy chain. The change from baseline in Neurofilament light chain will be analyzed using a mixed effects model as described under II.

### Interim analyses {21b}

We formalized one interim analysis with the ability to stop for superiority or futility, which will be conducted at 57% of the required number of events. The following stopping rules are defined (Table 2):

**Table 2:** Pre-determined stopping rules

Analysis	% of events	Futility		Superiority	
		*Z*	*Beta-spend*	*Z*	*Alpha-spend*
**I**	57%	0.36	0.0370	2.60	0.0046
**II**	100%	1.99	0.1630	1.99	0.0204
**Total**			**0.20**		**0.025**

The pre-specified stopping rule for efficacy is be based on the Lan-DeMets procedure with O’Brien-Fleming alpha-spending function with an asymmetric two-sided group sequential design with 80% power and 2.5% type I error. Upper bound spending computations assumed that the trial will continue if the lower bound is crossed (i.e., non-binding). At the interim analysis, a standardized likelihood ratio test statistic is calculated and compared to the *Z*-values provided in the table above. As soon as the test-statistic falls outside either the futility or superiority boundary, the study may be stopped for either superiority or futility.

In case enrolment is slow or a stopping rule is crossed early, the trial is likely to be terminated before all 171 patients have been enrolled. If treatment is futile, the trial can be stopped on average after 28.0 months since first enrolled patient with an average sample size of 137 patients. In this scenario, the probability for individual patients to remain in the trial for 18 months or more is 11.3% and to complete 24 months is 1.8%.

### Methods for additional analyses (e.g., subgroup analyses) {20b}

Subgroup analyses for the primary endpoint will be carried out separately for two predefined subgroups in the same manner as described in the relevant section.

These subgroups are:

- Utrecht stage: early vs. late (<0.25 vs. >0.25).
- Neurofilament light chain: high vs. low.

### Methods in analysis to handle protocol non-adherence and any statistical methods to handle missing data {20c}

Missing data in any of the outcomes will not be imputed.

### Plans to give access to the full protocol, participant level-data and statistical code {31c}

Non-identifiable patient-level data may be available on request.

## Oversight and monitoring

### Composition of the coordinating center and trial steering committee {5d}

The UMC Utrecht is the coordinating center, but coordination of the trial and of trial sites is performed by the sponsor, TRICALS. There are weekly meetings with the lead investigator, the medical reviewer, the project manager and clinical research associate. The latter two are also part of the sponsor safety committee. There is no trial steering committee, as tasks of the trial steering committee have been delegated to the Data Safety Monitoring Board (DSMB).

### Composition of the data monitoring committee, its role and reporting structure {21a}

A DSMB has been established to provide oversight of safety and efficacy considerations during the conduct of the current study and to provide advice to the sponsor regarding actions the committee deems necessary for the continued protection of patient enrolled in the study. The DSMB is an independent multidisciplinary group that, collectively, has experience in the management of patients with ALS, and in the conduct and monitoring of randomized clinical trials. DSMB members are independent of the sponsor and all investigational study sites.

The DSMB will provide recommendations about stopping or continuing the trial. To contribute to enhancing the integrity of the trial, the DSMB may also formulate recommendations relating to the selection, recruitment, and retention of patients, their management, improving adherence to protocol-specified regimens and retention of patients, and the procedures for data management and quality control. The DSMB may decide to prematurely terminate the trial for safety when the interim safety analysis indicates that serious adverse effects occur significantly more often in one group than in the other group, or when there is evidence for accelerated disease progression in the treatment arm compared to the placebo arm. Additionally, the DSMB may decide to prematurely terminate the trial for efficacy when any of the stopping rules as described previously are met.

The advice(s) of the DSMB will only be sent to the sponsor of the study. Should the sponsor decide not to fully implement the advice of the DSMB, the Sponsor will send the advice to the reviewing IRB, including a note to substantiate why (part of) the advice of the DSMB will not be followed.

### Adverse event reporting and harms {22}

Adverse events will be inquired for during each clinical visit and monitored using the LiSERS questionnaires when commencing the intervention. All adverse events reported spontaneously after the study medication has been provided (i.e., after baseline) by the patient or observed by the investigator or staff will be recorded. Due to an average delay of diagnosis of 1 year in Europe and an average life expectancy of 3 years for patients with ALS, a considerable proportion of the included ALS patients are expected to decease during the 2-year follow-up. These deaths are of natural disease progression and will not be reported as SAEs. All AEs will be followed until they have abated or until a stable situation has been reached. Depending on the event, follow up may require additional tests or medical procedures as indicated, and/or referral to the general physician or a medical specialist.

Expedited reporting of suspected unexpected serious adverse reactions (SUSARs) of immediate concern to patient safety will be done by the sponsor through the web portal EudraVigilance to the IRB; this suffices as notification to the competent authority. The remaining SUSARs will be recorded in an overview list accompanied by a brief report highlighting the main points of concern that will be submitted once every half year to the IRB.

The Sponsor will report expedited all SUSARs to the competent authorities in other member states, according to the requirements of the member states. The expedited reporting will occur not later than 15 days after the sponsor has first knowledge of the adverse reactions.

For fatal or life-threatening cases, the term will be maximal 7 days for a preliminary report with another 8 days for completion of the report. In addition to the expedited reporting of SUSARs, the sponsor will submit, once a year throughout the clinical trial, a safety report to the accredited IRB and competent authorities of the concerned member states.

This safety report consists of: a list of all suspected (unexpected or expected) serious adverse reactions, along with an aggregated summary table of all reported serious adverse reactions, ordered by organ system, per study, and a report concerning the safety of the patients, consisting of a complete safety analysis and an evaluation of the balance between the efficacy and the harmfulness of the medicine under investigation.

### Frequency and plans for auditing trial conduct {23}

The sponsor (or its authorized representative) has the obligation to follow this study closely to ensure that the study is conducted in accordance with the protocol, International Conference on Harmonisation (ICH) and GCP guidelines, national and international regulatory requirements, and the current Declaration of Helsinki throughout its duration, by means of personal visits to the investigator’s facilities and other communication. These visits will be conducted to evaluate the progress of the study, verify that the rights and well-being of the patients are protected, and verify the reported clinical study data are accurate, complete, and verifiable from source documents. This includes review of Informed Consent Forms, results of tests performed as a requirement for participation in this study, and any other medical records (e.g., laboratory reports, clinic notes, IP dispensing log, pharmacy records, patient-completed questionnaires, telephone logs, ECGs) required to confirm information contained in the eCRFs. The monitoring frequency during the study should be at least 2 visits per year per center, of which at least 1 on-site visit. Staff should be available during these visits for discussion on the conduct of the study as well as to facilitate the review of the clinical study records and resolve/document any discrepancies found during the visit. All monitoring activities will be reported and archived. In addition, monitoring visits will be documented at the clinical site by signature and date on the study-specific monitoring log.

### Plans for communicating important protocol amendments to relevant parties (e.g., trial patients, ethical committees) {25}

Amendments are changes made to the research after a favorable opinion by the accredited IRB has been given. All substantial amendments will be notified to the IRB, as they requires IRB approval, and to the competent authority. A “substantial amendment” is defined as an amendment to the terms of the IRB application, or to the protocol or any other supporting documentation, that is likely to affect to a significant degree:

- The safety or physical or mental integrity of the patients of the trial;
- The scientific value of the trial;
- The conduct or management of the trial;
- The quality or safety of any intervention used in the trial; and
- The addition of a new experimental compound.

Non-substantial amendments will not be notified to the accredited IRB and the competent authority, but will be recorded and filed by the sponsor. Examples of non-substantial amendments are typing errors and administrative changes like changes in names, telephone numbers and other contact details of involved persons mentioned in the submitted study documentation.

### Dissemination plans {31a}

Results from the study will be submitted for publication by the investigators only, in international peer-reviewed medical journals.

## Discussion

In this study we aim to provide conclusive evidence about the life-prolonging potential of lithium carbonate in a genetic ALS subgroup. As such, we have defined the primary endpoint as a composite of respiratory free survival, which has been recommended by the FDA and EMA for clinical trials in ALS [50, 51]. Even though a minority of ALS patients will be eligible for participation in the current trial due to need of homozygosity for the C-allele at SNP rs12608932 in *UNC13A*, there are several aspects of the trial that will hopefully ensure prompt enrolment. The TRICALS infrastructure allows access to a large population of potential trial participants, as each site sees between 37 and 264 new ALS patients yearly, based on a feasibility questionnaire [7]. We expect that 75 to 80% of these patients would be eligible for genetic screening due to the use of the TRICALS risk-profile score described previously, which allows for more patients to enroll than when using conventional eligibility criteria [18]. Finally, the event-driven and group-sequential design overcomes potential limitations encountered in previous trials, and minimizes the exposure of patients to ineffective treatment arms [54].

The main risk associated with this study is the side-effects of lithium. As multiple trials with lithium carbonate have been performed in ALS, the side-effects of lithium carbonate in the target population of this study are well known, as well as pitfalls pertaining to its use [14, 15, 20]. One previous trial was discontinued due to extensive patient discontinuation rates, partially due to the high number of SAEs [20]. While the following placebo controlled trials reported no major safety concerns [14, 15], we aim to minimize the risk of lithium intoxication by excluding patients at increased risk, and by frequent monitoring of both the plasma lithium concentration as well as earlier detection of looming intolerability through the LiSERS questionnaire.

Recent advancements in the field of ALS therapeutics could possibly affect the conduct of this study, as new treatment options are either in development or in the process of market authorization. A combination of oral sodium phenylbutyrate and ursodoxicoltaurine has been found to delay the rate of progression in a 6-month study and has been approved recently by the Canadian health authorities [55]. Furthermore, positive results of genetic therapy, Tofersen, specifically aimed at mutations in *SOD1* have been published [56]. Both of these compounds are yet to be approved by the relevant authorities in countries where the current study takes place. Nevertheless, should registration follow, an amendment to the current protocol will be made to include sodium phenylbutyrate-ursodoxicoltaurine, and Tofersen as a concomitant therapies, where new patients are preferably on a stable dose 30 days prior to randomization.

Genetic testing is part of the study protocol and can be a challenging topic to discuss with patients [57]. As blinding to UNC13A status is impossible in the current study, results will be shared with patients. This leads to some considerations regarding genetic and prognostic counseling. The life-time risk for developing ALS is 1:350 for people from European descent; homozygosity for the C-allele at SNP rs12608932 adds an almost negligible increase from to 1.13 in 350 [58]. Furthermore, the C-allele at SNP rs12608932 is a common genetic polymorphism; approximately 14% of the total population are homozygous [58, 59]. It is, therefore, a risk factor for ALS, but does not cause ALS or confer the disease in a Mendelian manner. As such, there is no need to refer patients homozygous for the C-allele at SNP rs12608932 to a clinical geneticist for additional counseling or testing of family members.

Homozygosity for the C-allele at SNP rs12608932, however, does have a large effect on the disease phenotype. The median survival for patients homozygous for the C-allele in *UNC13A* is shorter by approximately six months, though UNC13A homozygous patients may still present with a slow disease course and long survival [60, 61]. In the ENCALS survival model, homozygosity for the C-allele at SNP rs12608932 was not an independent predictor of survival [19]. This is due to the fact that homozygosity for the C-allele at SNP rs12608932 is associated with clinical characteristics that have a larger survival effect such as a bulbar onset and a delayed age at onset [19, 61]. As such, the *UNC13A* genetic status does not add predictive information on top of what can be said based on clinical characteristics alone. It is part of standard clinical care to discuss prognosis with patients based on their clinical characteristics. This is of particular importance for those patients where the disease progression rate will likely be rapid as it directly affects clinical decisions such as weight management and respiratory support.

## Trial status

Protocol version 1.5, 06-04-2022. Recruitment has begun on the 9th of august 2021 and is expected to be completed at the end of 2024.

## Supplementary Information

**Additional file 1.**

## Data Availability

After completion, results of the trial will be presented in peer reviewed scientific journals. Any data required to support the protocol can be supplied on request.

## Abbreviations

AE: Adverse event
ALS: Amyotrophic lateral sclerosis
ALSFRS-R: ALS functional rating scale (revised)
ALS-FTD-Q: ALS Frontotemporal dementia questionnaire‘
ALT: Alanine aminotransferase
AST: Aspartate aminotransferase
AR: Adverse reaction
CMAP: Compound muscle action potential
DSMB: Data Safety Monitoring Board
DRI: Death or respiratory insufficiency
ECAS: Edinburgh Cognitive and Behavioural ALS Screen
ENCALS: European Network for the Cure for ALS
eCRF: Electronic Case Report Form
eGFR: Estimated glomerular filtration rate
EudraCT: European drug regulatory affairs Clinical Trials
GCP: Good Clinical Practice
IC: Informed consent
LiSERS: Lithium Side Effects Rating Scale
NIV: Non-invasive ventilation
QALY: Quality adjusted life year
RCT: Randomized controlled trial
SVC: Slow Vital Capacity
SUSAR: Suspected unexpected serious adverse reaction
TRICALS: TRICALS is the first international research initiative uniting patients, top researchers and ALS foundations to reach our one goal: find an effective treatment for ALS
WOCBP: Women of Child-Bearing Potential
ULN: Upper limit of normal
