# Supplementary material for: Lithium carbonate in amyotrophic lateral sclerosis patients homozygous for the C-allele at SNP rs12608932 in UNC13A: protocol for a confirmatory, randomized, group-sequential, event-driven, double-blind, placebo-controlled trial
Source: Trials. 2022 Dec 5;23:978. doi: 10.1186/s13063-022-06906-5 (PMC9721045; doi:10.1186/s13063-022-06906-5)
Supplement: Supplementary file 1 — Additional file 1. [file 13063_2022_6906_MOESM1_ESM.pdf]

The Netherlands

Lead researcher: Prof. Dr. L.H. van den Berg
UMC Utrecht
Heidelberglaan 100
3584 CX, Utrecht, The Netherlands

Belgium

Lead researcher: Dr. Philip van Damme
University Hospital Leuven
Herestraat 49
3000, Leuven, Belgium

Ireland

Lead researcher: Prof. Dr. Orla Hardiman
Beaumont Hospital
Beaumont Rd, Beaumont, Dublin, Ireland

Sweden

Lead researcher: Dr. Caroline Ingre
Karolinska University Hospital
Eugeniavägen 3
171 64 Solna Stockholm, Sweden

United Kingdom

Lead researcher: Prof. Dr. Ammar Al-Chalabi
King's College Hospital
Bessemer Road
SE5 9RS, London, UK

Lead researcher: Dr. Thomas Lambert
University Hospitals of North Midlands NHS Trust
Newcastle Road
ST4 6QG, Stoke-on-Trent, United Kingdom

Lead researcher: Prof. Chris McDermott
Sheffield Teaching Hospitals NHS Foundation trust

Herries Road
S5 7AU, Sheffield, United Kingdom

Lead researcher: Dr. Suvankar Pal
University of Edinburgh
49 Little France Crescent
EH16 4SB, Edinburgh, United Kingdom

Lead researcher: Dr. Andrea Malaspina
University College London Hospital NHS
Queen Square
WC1N 3BG, London, United Kingdom

Australia
Lead researcher: Prof. Dr. Matthew Kiernan
The University of Sydney (Royal prince Alfred hospital)
94 Mallett Street Camperdown
NSW 2050 Sydney, Australia

Lead researcher: Prof. Steve Vucic
Concord hospital Sydney
Hospital Rd
Concord, Sydney, NSW 2139, Australia

Lead researcher: Associate Professor Robert Henderson
Royal Brisbane and Women's Hospital
Butterfield St
Brisbane, QLD 4029, Australia

Lead researcher: Dr. David Schultz
Flinders Medical Centre
Flinders Dr
Adelaide, SA 5042, Australia

Lead researcher: Dr. Susan Mathers & Prof Paul Talman
Calvary Health Care Bethlehem152 Como Parade
West Parkdale VIC 3195, Australia

Lead Researcher: Prof. Merilee Needham

Perron Institute

8 Verdun St

Nedlands, Perth, WA 6009, Australia

Spain

Lead researcher: Prof. Dr. Monica Povedano

Bellvitge University Hospital

Carrer de la Feixa Llarga, s/n

08907, Barcelona, Spain
